# Tailoring PBT Performance Through PBT/POE-g-GMA Nanocomposites with MWCNT

**DOI:** 10.3390/polym17212962

**Published:** 2025-11-06

**Authors:** Eduardo da Silva Barbosa Ferreira, Elieber Barros Bezerra, Carlos Bruno Barreto Luna, Edson Antonio dos Santos Filho, Renate Maria Ramos Wellen, Edcleide Maria Araújo

**Affiliations:** 1 Department of Materials Engineering, Federal University of Campina Grande, Campina Grande 58429-900, Brazil; 2 Department of Materials Engineering, Federal University of Paraíba, João Pessoa 58051-900, Brazil

**Keywords:** PBT, POE-g-GMA, carbon nanotubes, electrical conductivity, nanocomposites

## Abstract

The production of polymer nanocomposites from supertough blends reinforced with carbon-based nanofillers has garnered attention in recent years due to improvements in their mechanical, thermal, and electrical properties. Currently, the main challenge is to develop materials with balanced performance for diverse industrial demands. In this context, this work aimed to produce nanocomposites of poly(butylene terephthalate) (PBT) and poly(ethylene-octene) grafted with glycidyl methacrylate (POE-g-GMA), reinforced with carbon nanotubes (MWCNTs). The PBT, the PBT/POE-g-GMA blend, and the respective MWCNT nanocomposites were initially premixed in an internal mixer and then processed in a co-rotational twin-screw extruder. After processing, they were injection-molded to obtain tensile, impact, and HDT test specimens. Mechanical (tensile, impact, and Shore D hardness), thermal (differential scanning calorimetry—DSC), thermomechanical (heat deflection temperature—HDT), electrical resistivity/conductivity, morphology, and Fourier transform infrared spectroscopy (FTIR) properties were evaluated. The results demonstrated a good balance among the investigated properties, with improvements in mechanical, thermal, and thermomechanical properties when compared to PBT. The impact strength of the nanocomposites reached 186 J/m, approximately 158% higher than that of neat PBT. The HDT reached approximately 55 °C in the PBT/POE-g-GMA/MWCNT5 nanocomposites, while the crystallization temperature increased by 11 °C, as evidenced by DSC, an aspect of great relevance for industrial applications. Furthermore, the PBT/POE-g-GMA/MWCNT5 nanocomposites exhibited an electrical conductivity of 1.06 × 10^−7^ S/cm, indicating potential for electrical applications.

## 1. Introduction

The growing demand for high-performance materials with specific properties for specific applications has sparked interest in polymer nanocomposites in both industry and academia. The development of these materials with enhanced characteristics is an important strategy in materials science and engineering, enabling the acquisition of unique properties through the incorporation of small amounts of nanofillers, thanks to their high surface area [1]. Among the most commonly used nanofillers in polymer matrices are clays, carbon nanotubes, cellulose nanofibers, metal oxides, and graphene [2,3]. Due to their nanoscale size and high specific surface area, these fillers are added to polymers to improve mechanical, thermal, and barrier properties and, in the case of conductive nanofillers, to increase electrical conductivity [4,5,6,7,8,9,10,11].

Among the polymers widely used for the production of blends and nanocomposites, poly(butylene terephthalate) (PBT) stands out as a semicrystalline thermoplastic with properties that make it attractive for various applications. These properties include a high crystallization rate, chemical resistance, dimensional stability, thermal resistance, and ease of processing, ensuring wide applicability in sectors such as automotive, packaging, electronics, and consumer goods [1,12,13,14,15,16]. Its high crystallization rate enables fast injection molding cycles, resulting in increased productivity and process savings [14]. However, PBT has weaknesses, such as low impact strength and a propensity for crack propagation [1,14]. To overcome these limitations, modification strategies are often used, including the formation of polymer blends with the addition of impact modifiers [17,18]. An effective example is the incorporation of poly(ethylene-octene) grafted with glycidyl methacrylate (POE-g-GMA), which acts as an elastomeric phase dispersed in the rigid matrix, promoting a significant improvement in toughness by absorbing and dissipating energy during fracture [19,20].

Furthermore, with advances in nanotechnology, it has become possible to enhance polymers through the incorporation of nanofillers, which, even at low concentrations, can produce significant changes in the material’s properties. Among the various nanofillers, multi-walled carbon nanotubes (MWCNTs) stand out for their high mechanical strength, excellent electrical and thermal conductivity, and high aspect ratio [21,22,23]. These characteristics confer a set of unique properties to the obtained nanocomposites, making them especially suitable for applications requiring electrostatic dissipation, shielding against electromagnetic interference, and greater thermal stability [24,25,26].

Shang et al. [19] produced supertough PBT/POE blends compatibilized with POE-g-GMA by extrusion. The impact strength increased by 18 times with the addition of 10% POE-g-GMA compared to neat PBT. Furthermore, SEM observations revealed that the addition of the compatibilizer effectively enhances the interaction between PBT and POE. In a related study, Zong et al. [27] obtained PBT/POE-g-GMA/PP/MWCNT nanocomposites prepared by melt blending. The nanocomposites obtained in this study have excellent electrical properties. Additionally, it was observed that increasing the content of MWCNTs and POE-g-GMA in the PBT/PP blend refines the morphology, thus influencing the conductive path of MWCNTs.

Thus, studies using PBT blends with impact modifiers have been explored, in addition to the use of nanofillers to enhance specific properties. However, the strategy proposed in this work presents a more comprehensive approach. PBT/POE-g-GMA blend nanocomposites were produced and the effect of different amounts of carbon nanotubes (MWCNTs) on the blend properties was evaluated, also comparing them with neat PBT. Therefore, this study aimed to obtain PBT/POE-g-GMA blend nanocomposites reinforced with different MWCNT contents (1–5 phr), evaluating the thermal, mechanical, thermomechanical, and morphological properties of the materials. This multifunctional approach allows for the direct correlation of structure, morphology, and final performance, providing a more complete understanding of the influence of MWCNTs on the PBT/POE-g-GMA blend. Thus, compared to some of the works in the literature, which generally focus on a limited number of properties or specific specifications, the present study advances to systematically evaluate the effect of nanofiller content on multiple properties, exploring the development of materials with a balance between mechanical, thermal and electrical performance.

## 2. Methodology

### 2.1. Materials

Poly(butylene terephthalate) (PBT), commercially known as Crastin S600F10 NC010, supplied by DuPont, in the form of granules, with a melt flow rate (MFR) of approximately 10.1 g/10 min (250 °C/2.16 kg), was used. Poly(ethylene-octene) grafted with 0.8% glycidyl methacrylate (POE-g-GMA), under the commercial name Coace W5B, was used as the elastomeric phase, produced by Xiamen Coace Plastic Technology (Xiamen, China), with a density of 0.91 g/cm^3^ and MFR between 3 and 8 g/10 min. Multi-walled carbon nanotubes (MWCNTs), supplied by Gelon under the trade name CNT-3080, with a diameter of 30–80 nm, a length of less than 20 µm, a specific surface area of 80–120 m^2^/g, and purity greater than 99%, were used as a nanofiller.

### 2.2. Obtaining Polymeric Nanocomposites

Initially, PBT, POE-g-GMA, and MWCNTs were dried in a vacuum oven for 24 h at 60 °C. Subsequently, a premix of the PBT/POE-g-GMA/MWCNTs formulations was performed, as shown in Table 1, using an internal mixer equipped with roller rotors (Rheomix 3000, Thermo Fisher Scientific (Waltham, MA, USA)), operating at 250 °C and 60 rpm for 3 min. The obtained material was subsequently ground in a knife mill. To ensure better dispersion of the nanocomposite components, the mixture was processed in a co-rotational twin-screw extruder, equipped with specific mixing elements, operating at 250 rpm, a feed rate of 3 kg/h, and a temperature profile of 220–225–225–230–235–235–240 °C. The extruded compounds were then injection molded in an Arburg Allrounder 207C Golden Edition injection molding machine (Radevormwald, Germany). Specimens were produced for tensile, impact, and HDT testing, in accordance with ASTM D638 (Type I), ASTM D256, and ASTM D648, respectively. The injection process was conducted at a mold temperature of 50 °C and a thermal profile of 240 °C in the first two zones, 245 °C in the third and fourth zones, and 250 °C in the final heating zone.

**Figure 1:**
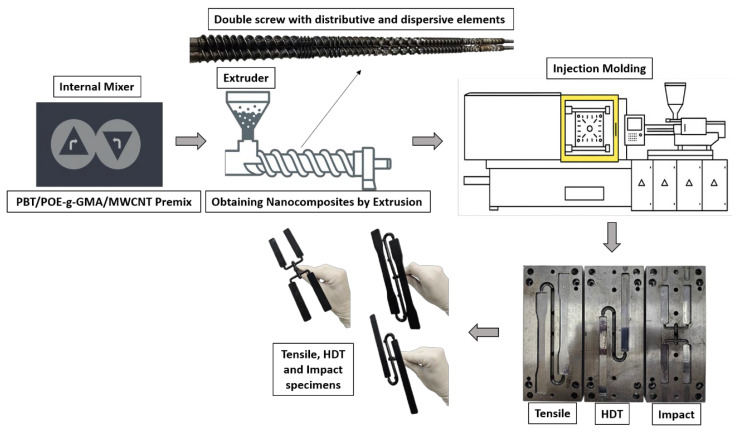
Processing scheme of polymeric nanocomposites.

The material obtained from the extrusion process was also used to prepare thin films for electrical resistivity and conductivity measurements. The films were produced using a hydraulic press (MH Equipamentos LTDA (Guarulhos, Brazil) operated at approximately 230 °C, under an initial pressure of 2 tons for 2 min, followed by a final pressure of 5 tons for 4 min. The processing scheme for obtaining the materials is presented in Figure 1.

## 3. Characterizations

### 3.1. Differential Scanning Calorimetry (DSC)

Thermal analyses were performed by DSC on a Shimadzu DSC-60Plus equipment (Kyoto, Japan). The procedure consisted of a heating, cooling, and reheating cycle, followed by a 2 min isotherm. Samples of approximately 3 mg were used and analyzed over a temperature range of 25 to 200 °C, with a heating rate of 10 °C/min. Throughout the measurement, a nitrogen atmosphere (50 mL/min) was maintained to ensure an inert environment. The degree of crystallinity (*X_c_*) of the samples was determined according to Equation (1) [28].

(1) $$X_{c}\left(\%\right)=\frac{{∆H}_{m}\;}{W\times {∆H}_{m}^{0}}\times 100$$

∆*H_m_* is the fusion enthalpy of PBT; ${∆H}_{m}^{0}$ is the fusion enthalpy, for 100% crystalline PBT, being 140 J/g [29] and *W* is the PBT content present in the system.

### 3.2. Fourier Transform Infrared Spectroscopy (FTIR)

Fourier transform infrared spectroscopy (FTIR) analyses were conducted using a BRUKER Alpha II spectrometer (Billerica, MA, USA), scanning in the range of 4000 to 450 cm^−1^.

### 3.3. Torque Rheometry

The torque rheometry test was used to evaluate evidence of chemical reaction during the processing of the PBT/POE-g-GMA blend. PBT, POE-g-GMA, and the PBT/POE-g-GMA blend were processed at 250 °C and 60 rpm for 10 min using an internal mixer equipped with roller rotors (Rheomix 3000, Thermo Fisher Scientific).

### 3.4. Izod Mechanical Impact Strength Test

Izod impact strength tests were performed on notched specimens using a 5.5 J pendulum in a Ceast Resil 5.5 instrument (Turin, Italy), following the procedures of ASTM D256-97. For each formulation, the results correspond to the average of 10 samples, obtained at room temperature. The impact strength test specimens, as observed in Figure 1, have an average length of 62.5 mm, a width of 12.7 mm, and a thickness of 3.2 mm. The notches, approximately 2.5 mm deep, were made on a Ceast Notschvis carving machine (Turin, Italy) in a “V” shape.

### 3.5. Mechanical Tensile Test

Tensile tests were conducted in accordance with ASTM D638 to evaluate the mechanical properties of the specimens. Measurements were taken on an Oswaldo Filizola BME (São Paulo, Brazil) equipped with a 20 kN load cell, using a deformation rate of 5 mm/min, at room temperature. For each composition, the results correspond to the average of 10 samples.

### 3.6. Shore D Hardness

The penetration resistance of the nanocomposites and blends was evaluated according to ASTM D2240 standard, using a Shore Type D durometer (Metrotokyo, São Paulo, Brazil). The test was performed with a 50 N load, applied by calibrated springs and standardized indenters. The results were obtained from the average of five measurements per sample.

### 3.7. Scanning Electron Microscopy (SEM)

Impact fracture surface of PBT, blends, and nanocomposites was examined by scanning electron microscopy (SEM) using the equipment Tescan FEG Mira 4 microscope (Brno, Czech Republic). Images were acquired at a voltage of 5 kV. To reduce charge buildup during analysis, the samples were previously coated with a thin layer of gold.

### 3.8. Heat Deflection Temperature (HDT)

The heat deflection temperature (HDT) of the materials was determined according to ASTM D648 standard, using a Ceast HDT 6 VICAT/N 6921.000 equipment (Turin, Italy). The tests were conducted by applying a stress of 1820 kPa and a heating rate of 120 °C/h (method A). Measurements of the test specimens, on average: thickness of 3.2 mm, width of 12.5 mm, length of 123.8 mm. The HDT was recorded when a deflection of 0.25 mm occurred, averaged over three specimens per formulation.

### 3.9. Electrical Resistivity and Conductivity

The electrical resistivity of films produced from PBT, blends, and nanocomposites was evaluated using a Keithley 8009 electrometer (Keithley Cleveland, OH, USA), applying the volumetric method as specified in ASTM D257. The electrical conductivity of the materials was calculated from the measured resistivity values. The thickness of the films tested was 1 ± 0.1 mm, the test voltage was 1 V, and the current was 20 mA. The system uses electrodes in the geometry of concentric disks, with a diameter of 5.4 cm. It has an upper electrode, which comes into direct contact with the upper surface of the sample; and the lower disk, divided into two concentric parts, the central electrode (also a circular disk), aligned with the upper electrode, and the annular electrode, to isolate the surface current.

The ohmic behavior curve (I–V) was obtained according to Equation (2):

(2) $$I=\;\frac{VA\;}{\rho L}\;$$

*I* is the electric current (amperes), *V* the applied voltage (V), *A* the electrode area (m^2^), ρ the resistivity of the material (Ω·m) and *L* the film thickness (or the distance between the electrodes).

## 4. Results and Discussion

### 4.1. Differential Scanning Calorimetry (DSC)

**Figure 2:**
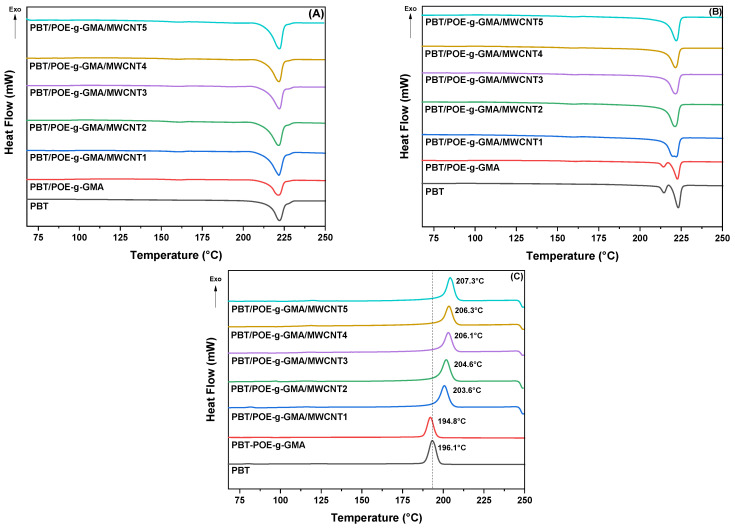
DSC curves for the first heating (**A**), second heating (**B**) and cooling (**C**) of the studied materials.

The DSC heating and cooling curves, together with the melting and crystallization parameters of PBT, the PBT/POE-g-GMA blend, and the studied nanocomposites, are shown in Figure 2A–C and Table 2, respectively. For the first heating (Figure 2A), a single endothermic peak, characteristic of PBT melting, can be observed for all samples, around 222 °C, indicating that the processing conditions resulted in a relatively homogeneous crystalline morphology. However, after the first cycle and elimination of the thermal history of the samples, a double melting peak appears in the second heating (Figure 2B), with endothermic peaks for neat PBT, at 214.8 °C and 223.2 °C, characteristic of crystal melting [30]. The same occurs for the PBT/POE-g-GMA blend, with a slight shift toward lower temperatures, producing values of 214.3 °C for T_m1_ and 222.6 °C for T_m2_.

The appearance of this double peak is associated with the formation of crystalline populations with different lamellar thicknesses during cooling. Furthermore, it may be associated with recrystallization and lamellar reorganization processes that occur after initial melting. When the material is completely melted during the first heating and then cooled in a controlled manner, the polymer chains have greater freedom to reorganize, forming lamellae of varying degrees of perfection. Thus, the melting of thinner lamellae occurs at lower temperatures, while the thicker, more ordered lamellae melt at higher temperatures, giving rise to the double peak. Other studies have also confirmed the presence of a double melting peak in neat PBT samples [31]. With the addition of MWCNTs, the double peak is absent, indicating that they reduce the occurrence of heterogeneous recrystallization. Thus, this suggests that MWCNTs act as a nucleating agent, promoting more uniform crystallization, limiting the formation of lamellar populations with varying degrees of perfection and, consequently, preventing the appearance of the double peak observed in PBT and the PBT/POE-g-GMA blend. This behavior was also observed in the degree of crystallinity of the materials, calculated from Equation (1). With the addition of different MWCNT contents, the degree of crystallinity increases, starting at 24.5% and 20.4% for PBT and the PBT/POE-g-GMA blend, reaching values of up to 38.9% for the PBT/POE-g-GMA/MWCNT nanocomposite.

The cooling curves (Figure 2C) show exothermic peaks for all compositions, characteristic of the PBT crystallization temperature. A tendency for the crystallization temperature to increase is observed in the nanocomposites obtained, ranging from 196.1 °C for neat PBT to 207.3 °C for the composition with 5 phr of MWCNT. This indicates that the MWCNT accelerated PBT crystallization, facilitating crystal nucleation. Furthermore, this increase in crystallization temperature is important from a production/industrial perspective, as injection molding cycles would require less time and energy for crystallization and the production of various parts for consumer sectors.

### 4.2. Fourier Transform Infrared Spectroscopy (FTIR)

**Figure 3:**
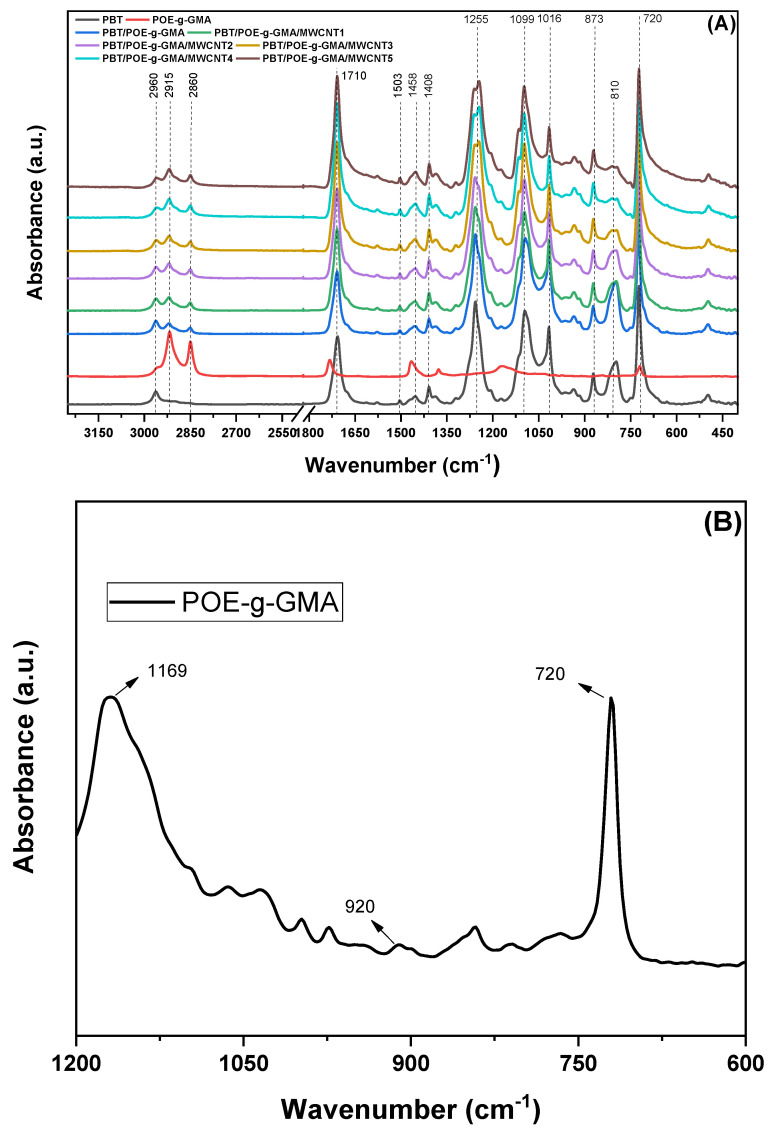
FTIR spectra of the studied compositions (**A**) and FTIR spectrum of POE-g-GMA at 1200–600 cm^−1^ (**B**).

Figure 3 shows the FTIR spectra of neat PBT, POE-g-GMA, PBT/POE-g-GMA blend, and nanocomposites with 1–5 phr MWCNT contents. For neat PBT, more intense absorption bands can be observed around 2960 cm^−1^, characteristic of asymmetric and symmetric stretching vibrations in the C–H bonds of the CH_2_ group; at 1710 cm^−1^, corresponding to the stretching of the C=O group; at 1503 cm^−1^, CH_2_ bending of the butylene group unit; the bending vibrations of the C–H bonds are located at 1408 and 1458 cm^−1^; the bands corresponding to the C–O and C–O–C stretching vibrations are identified at 1255 and 1099 cm^−1^, respectively. The bands at 1018, 873, and 720 cm^−1^ in the PBT spectrum can be attributed to the aromatic ring. Around 810 cm^−1^, a characteristic band of the C–H bending of the terephthalic unit is observed [32,33]. As for POE-g-GMA, as observed in Figure 3A and for better visualization of the region between 1200 and 600 cm^−1^, as shown in Figure 3B, it is possible to observe absorption bands at 2915 and 2860 cm^−1^, corresponding to the CH_3_ symmetric stretching. From Figure 3B, it is possible to observe, for POE-g-GMA, bands present at 1169, 920, and 720 cm^−1^, referring to the C–O symmetric stretching, out-of-plane deformation of the C–O–C bond of the epoxy ring present in GMA, and deformation of the CH_2_ group, respectively [34,35].

It is observed that, with the PBT/POE-g-GMA blend, more intense peaks appear at 2915 and 2860 cm^−1^, due to the addition of the impact modifier to the PBT matrix. Furthermore, there is a decrease in intensity, as well as the appearance of a small shoulder, when comparing neat PBT and the PBT/POE-g-GMA blend, in the bands 1710 and 1255 cm^−1^, which may be indicative of a possible reaction, leading to significant gains in impact strength, as observed later.

**Figure 4:**
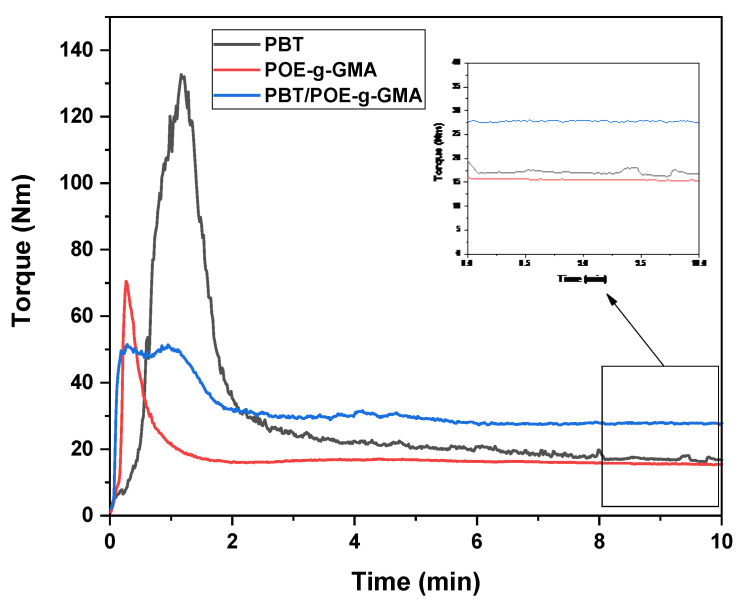
Torque curves as a function of time for PBT, POE-g-GMA, and the PBT/POE-g-GMA blend.

The torque rheometry rheological test was performed to confirm the reaction between PBT and POE-g-GMA. Torque vs. time curves were obtained for PBT, POE-g-GMA, and the PBT/POE-g-GMA blend, as shown in Figure 4. The torque-time graph shows that the PBT/POE-g-GMA blend exhibits a distinct behavior compared to the pure components, with a pronounced increase and stabilization at values higher than those observed for PBT and POE-g-GMA. This increase in torque in the PBT/POE-g-GMA blend suggests an increase in the material’s viscosity, which suggests the occurrence of reactions between the epoxy groups of GMA present in POE-g-GMA and terminals groups of PBT during processing. This rheological evidence corroborates the results obtained by FTIR.

### 4.3. Izod Mechanical Impact Strength Test

**Figure 5:**
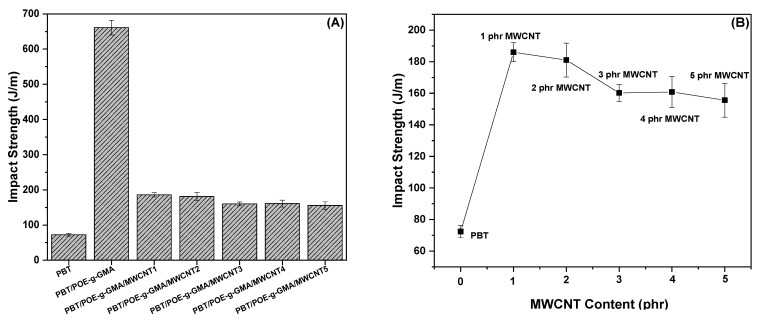
Izod impact strength of the studied materials (**A**) and comparison of nanocomposites with neat PBT (**B**).

Figure 5 shows the Izod impact strength results for neat PBT, the PBT/POE-g-GMA blend with the addition of 15% POE-g-GMA impact modifier, and PBT/POE-g-GMA/MWCNT nanocomposites with MWCNT contents ranging from 1 to 5 phr. It can be observed that PBT has the lowest impact strength value among the studied compositions, with a value of 72.4 J/m. Low impact strength values for neat PBT, when notched, have also been observed in the literature [19,32,36]. Furthermore, Tomar and Maiti [32] demonstrated that PBT exhibits a high crack initiation energy and low crack propagation energy, resulting in high impact strength when not notched, but low impact strength when notched. With the addition of POE-g-GMA, there is a considerable increase in impact strength when compared to neat PBT, reaching values close to 660 J/m. The significant increase in the system’s impact strength is attributed to the addition of POE elastomeric particles and the action of GMA between the PBT and POE phases, which improves interfacial adhesion and results in a more efficient material under impact. Furthermore, the increased toughness observed in materials containing POE-g-GMA is also related to dispersed phase cavitation and matrix fibrillation, mechanisms by which the dispersed phase absorbs a large amount of energy, thus increasing the materials’ impact strength. Shang et al. [37] observed that higher POE-g-GMA contents (up to 30%) in PBT/POE-g-GMA blends significantly increase the system’s impact strength. However, a marked reduction in tensile strength was observed compared to neat PBT, which may limit the material’s use in certain applications.

For PBT/POE-g-GMA/MWCNT nanocomposites, impact strength dropped sharply for all five concentrations, reaching values of 186 to 155 J/m compared to the PBT/POE-g-GMA blend. This decrease occurs because the carbon nanotube acts as a rigid filler, reducing the mobility of the POE-g-GMA elastomeric phase. With the formation of polymeric nanocomposites, the addition of MWCNTs, especially the poorly dispersed and agglomerated ones, as observed in the SEM will partially inhibit these toughening mechanisms, favoring crack initiation and a decrease in impact resistance, when compared with the PBT/POE-g-GMA blend. Notably, as shown in Figure 5B, even with this sharp decline, the nanocomposites still yielded excellent results compared to neat PBT. Impact strength increased by 158%, as shown in Figure 5B, which is significant from both scientific and technological perspectives.

### 4.4. Mechanical Tensile Test

**Figure 6:**
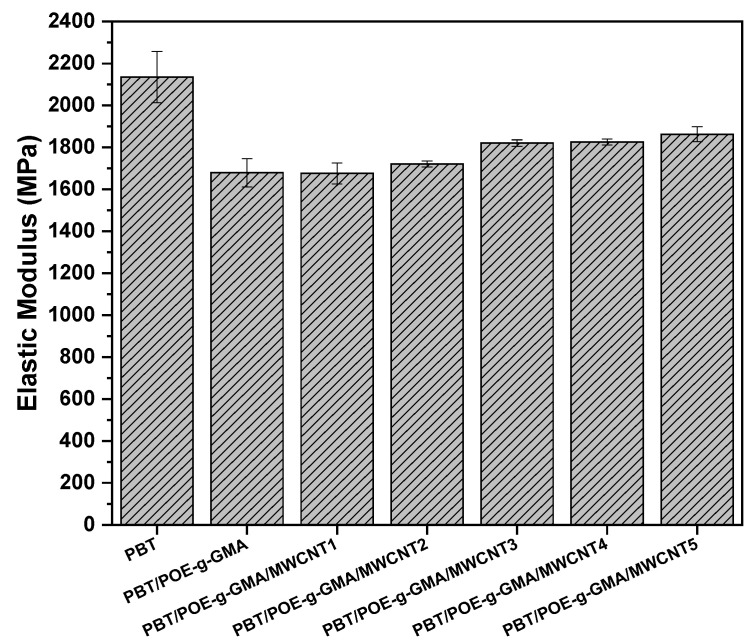
Tensile elastic modulus of the studied materials.

**Figure 7:**
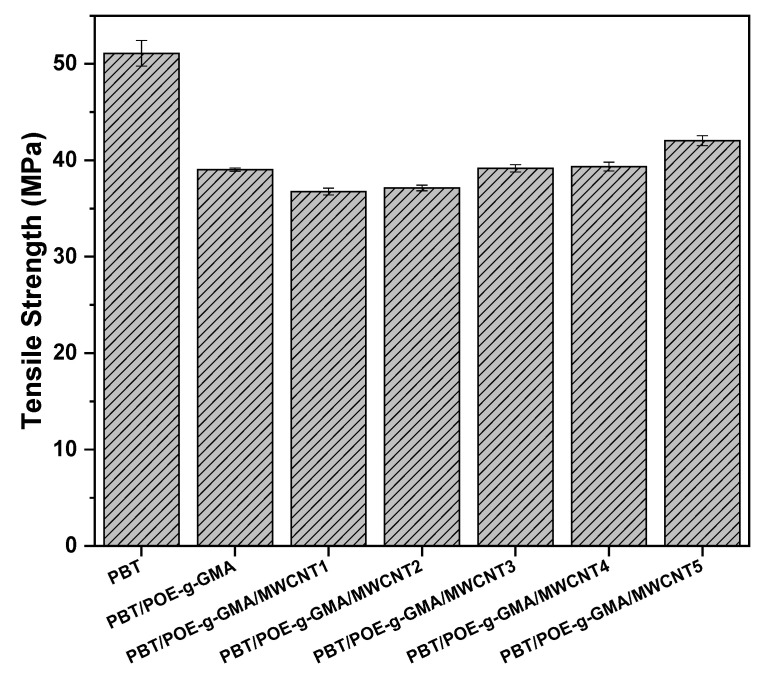
Tensile strength of the studied materials.

The results of the mechanical properties of the tensile test are shown in Figure 6 and Figure 7. It is possible to observe, for the elastic modulus results (Figure 6), that the neat PBT presents the highest result among the compositions studied, with a value of around 2135 MPa, characteristic of a material with high stiffness. Mane et al. [38] studied PBT blends incorporated with polycarbonate (PC) and observed similar results for the elastic modulus for the neat PBT. With the addition of POE-g-GMA, there is a pronounced decay in the elastic modulus of the material, with a value of 1678 MPa, characteristic of the addition of an elastomeric material to the PBT matrix, decreasing the stiffness of the material when compared to the neat PBT. This decrease is in agreement with the Shore D hardness results, observed later. With the addition of carbon nanotubes, the materials’ elastic modulus increased compared to the PBT/POE-g-GMA blend, particularly at higher concentrations, reaching values of approximately 1862 MPa for the PBT/POE-g-GMA/MWCNT5 nanocomposite. This increased elastic modulus is directly related to the rigid nature of the MWCNT, thus hindering the free mobility of the polymer chain.

Figure 7 shows the tensile strength results of the studied materials. A similar behavior to the elastic modulus can be observed, with PBT presenting the highest value among the analyzed materials, with a tensile strength of 51.1 MPa. With the addition of POE-g-GMA, a characteristic decay occurs, similar to the addition of an impact modifier to a rigid material, which requires a lower stress for material deformation to occur, resulting in approximately 39 MPa. It is possible to note that the addition of lower MWCNT contents to the PBT/POE-g-GMA blend results in a decrease in the tensile strength of the materials, with results of 36.7 and 37.1 MPa for the PBT/POE-g-GMA/MWCNT1 and PBT/POE-g-GMA/MWCNT2 nanocomposites, respectively. However, when higher levels of nanofiller are added, this effect is overcome, resulting in an increase in tensile strength, reaching 42 MPa for the PBT/POE-g-GMA/MWCNT5 composition. In fact, the addition of lower levels of MWCNT was insufficient for the nanofiller to act as reinforcement for the PBT/POE-g-GMA blend; however, with the addition of higher levels, this behavior was reversed. According to Zhu et al. [39], the mechanical performance of nanocomposites using MWCNT is directly associated with their morphology, specifically dispersion and distribution, resulting in improved mechanical properties.

**Figure 8:**
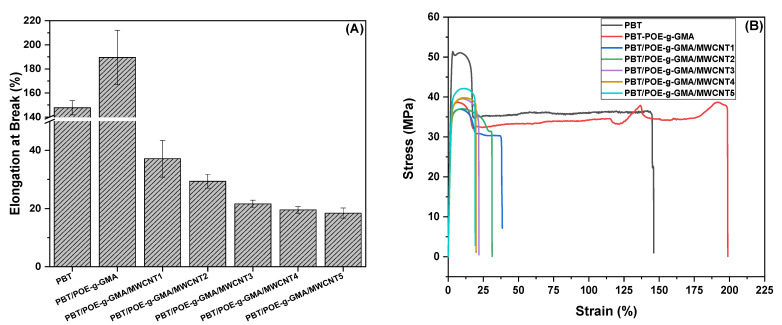
Elongation at break (**A**) and stress–strain curves (**B**) of the studied materials.

Figure 8 shows the elongation at break Figure 8A and stress–strain curves Figure 8B of the analyzed samples. PBT, although presenting the lowest impact strength, exhibits high elongation at the test speed used (5 mm/min), reaching approximately 147%. With the incorporation of 15% POE-g-GMA, elongation increases significantly to approximately 200%. This behavior can be attributed not only to the presence of the elastomeric component but also to the improved interfacial adhesion promoted by the GMA groups of the impact modifier. In the nanocomposites, as the MWCNT content increases, a progressive reduction in elongation is observed, due to the nanotubes acting as stress concentrators and partially interfering with the action of POE-g-GMA.

### 4.5. Shore D Hardness

**Figure 9:**
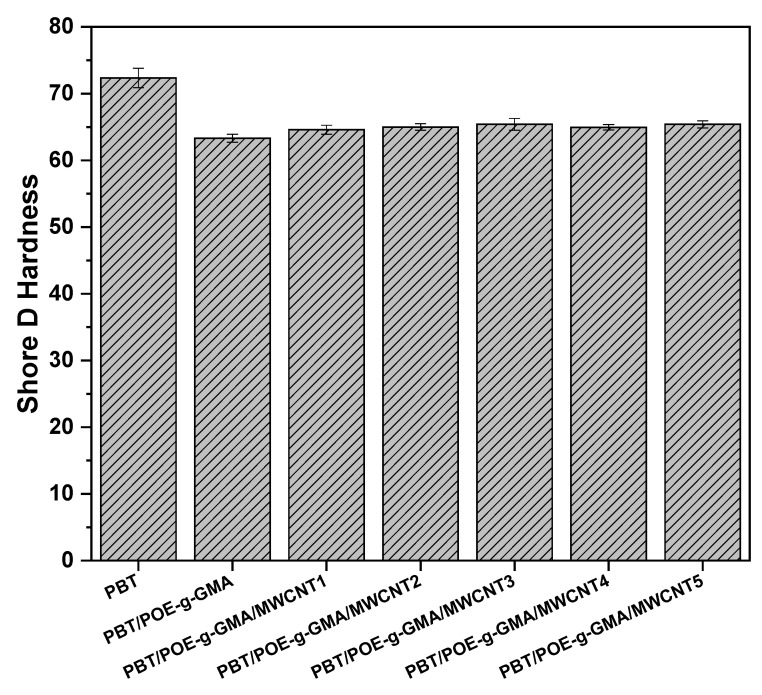
Shore D hardness of the studied materials.

Shore D hardness analysis is highly relevant to the polymeric materials industry, particularly in the evaluation of nanocomposites, as it provides information on surface penetration resistance and correlates stiffness with mechanical properties under impact and tensile stress. Figure 9 shows the Shore D hardness results for PBT, the PBT/POE-g-GMA blend, and the studied nanocomposites. PBT presents the highest Shore D hardness among the materials analyzed, with a value of 72.3 Shore D, characteristic of a rigid material, as observed in the impact and tensile strength results. This result is also reported in the literature [40]. With the addition of 15% POE-g-GMA, the surface hardness of the material decreases due to the elastomeric nature of the impact modifier used, reaching a Shore D hardness of 63.3, and thereby decreasing the surface strength of the material. The formation of PBT/POE-g-GMA/MWCNT nanocomposites, compared to the PBT/POE-g-GMA blend, promoted a slight increase in the surface hardness of the materials. Furthermore, a gradual increase was observed with the MWCNT content, reaching values of approximately 65.4 Shore D for the PBT/POE-g-GMA/MWCNT5 system, indicating an increase in the surface hardness of the materials, which corroborates the results of the elastic modulus (Figure 6).

### 4.6. Scanning Electron Microscopy (SEM)

**Figure 10:**
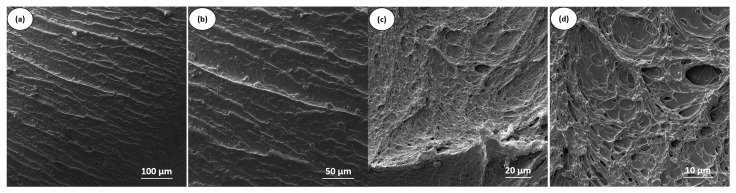
Fracture surface of the impact strength test obtained by SEM of neat PBT with 500× (**a**) and 1k× (**b**) and of the PBT/POE-g-GMA blend with 2k× (**c**) and 5k× (**d**).

Figure 10 shows the SEM micrographs of neat PBT and the PBT/POE-g-GMA blend, with different magnifications. For neat PBT (Figure 10a,b), a smoother surface is observed compared to the PBT/POE-g-GMA blend (Figure 10c,d), although with a certain degree of plastic deformation, resulting in approximately 72.4 J/m, as previously observed in Figure 5. Similar images were reported by Shin and Park [41]. With the addition of 15% POE-g-GMA, a greater roughness is evident on the fracture surface, characteristic of plastic deformation, cavitation of the elastomeric phase, and fibrillation of the matrix due to the flow process, a fact directly related to the increase in the material’s toughness, observed in the impact strength results [42].

**Figure 11:**
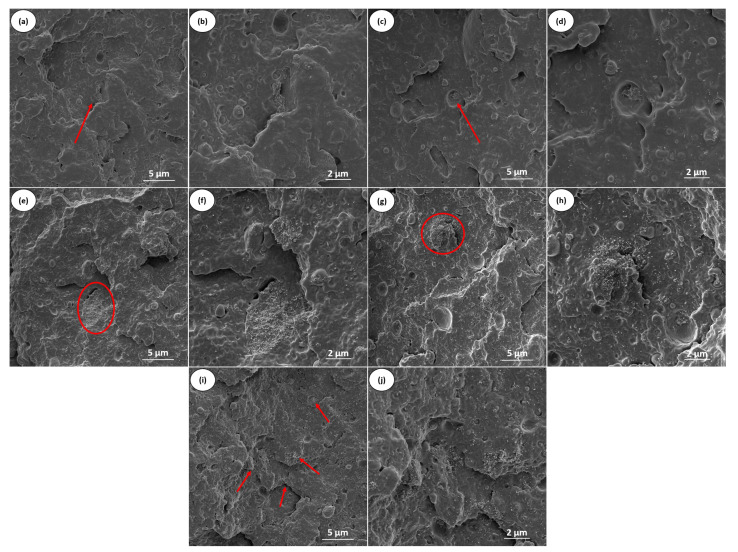
Fracture surface of the impact strength test obtained by SEM of the studied nanocomposites with 10k× and 20k×. PBT/POE-g-GMA/MWCNT1 (**a**,**b**); PBT/POE-g-GMA/MWCNT2 (**c**,**d**); PBT/POE-g-GMA/MWCNT3 (**e**,**f**); PBT/POE-g-GMA/MWCNT4 (**g**,**h**); PBT/POE-g-GMA/MWCNT4 (**i**,**j**).

With the addition of carbon nanotubes to the PBT/POE-g-GMA blend, a change in the fracture surface is observed. In addition to the presence of agglomerates on the fracture surface of the studied nanocomposites (red circles in Figure 11e,g), voids are also present in the structure, as well as more visible and detached particles from the POE-g-GMA. This is due to the MWCNTs being preferentially present in the dispersed phase. Furthermore, this fact restricts the cavitation capacity of the elastomeric phase, thus limiting the material’s toughness. Even with the decrease in the material’s toughness, the addition of up to 5 phr carbon nanotubes still results in a well-adhered POE-g-GMA to the PBT matrix, demonstrating a considerable gain in toughness compared to the impact strength results for neat PBT (see Figure 5). It can be noted, for the PBT/POE-g-GMA/MWCNT5 composition (Figure 11i,j), that the presence of MWCNT clusters presents an interconnected network on the surface (red arrows), unlike the lower MWCNT contents, which favor an electrical percolation network, as will be observed in later results.

**Figure 12:**
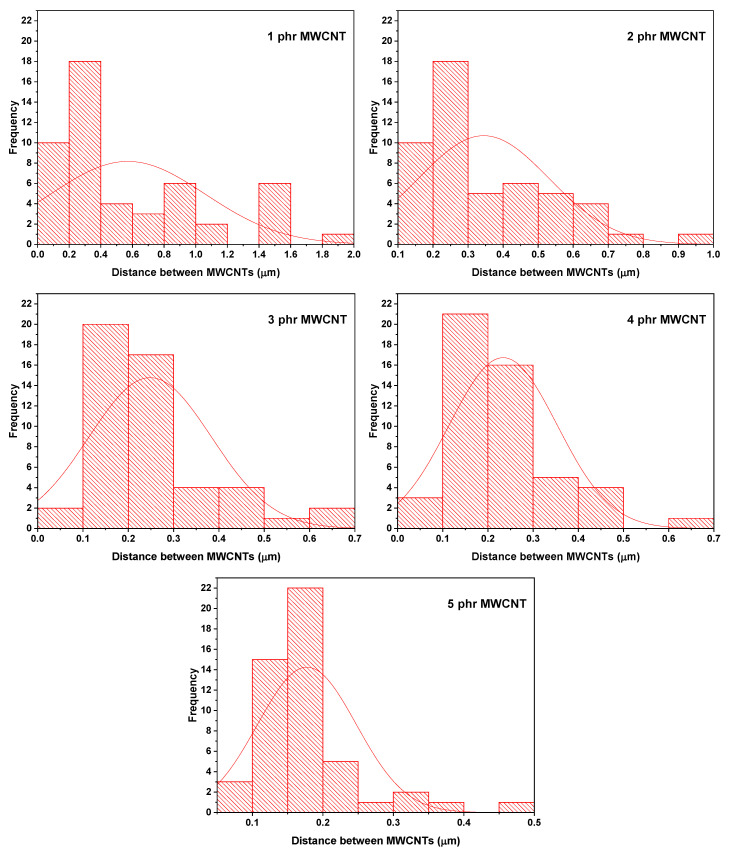
Distance between MWCNTs for nanocomposites studied.

Figure 12 shows the average distance between MWCNTs in the studied nanocomposites. The histograms show that the average distance between nanotubes progressively decreases as the MWCNT content increases from 1 to 5 phr. At the lowest contents (1–3 phr), the distributions show higher average distance values, indicating discontinuous dispersion, with regions containing few MWCNTs close to each other and large areas of the polymer matrix practically isolated from conductive charges. This microstructure does not favor the formation of conductive pathways, as explained by the later conductivity results. In the 4 phr samples, the average distances shift to smaller values, suggesting a progressive approximation between the nanotubes and the formation of conductive clusters. However, significant dispersion remains. Finally, at 5 phr, the distribution shows a peak at very small distances (<0.2 µm), indicating direct contact or sufficient proximity between the MWCNTs to form a continuous conductive network, resulting in an abrupt change in electrical conductivity (Table 3).

### 4.7. Heat Deflection Temperature (HDT)

**Figure 13:**
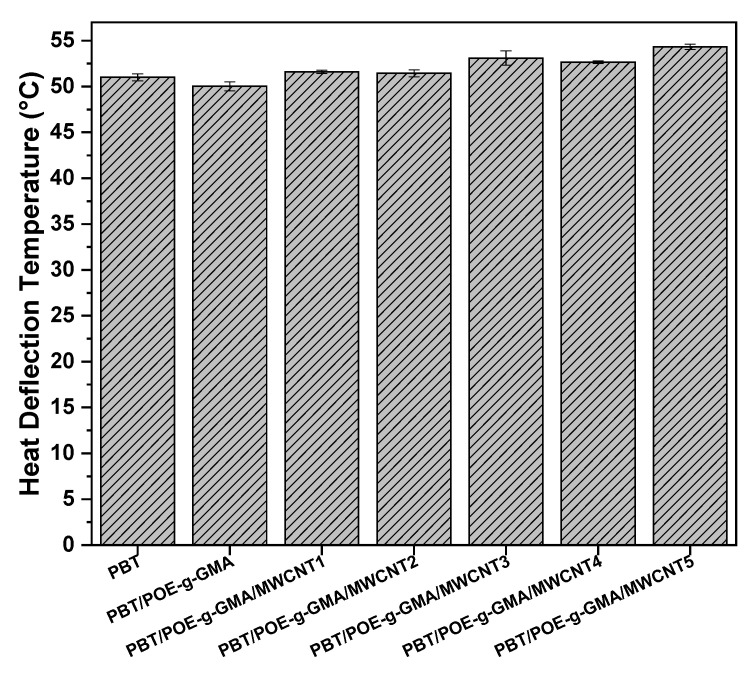
HDT of the studied materials.

According to Shen et al. [43], the test to obtain the heat deflection temperature (HDT) is a relevant parameter for analyzing the thermomechanical behavior of composite materials, serving as a reference for estimating the maximum application limit, according to the temperature, in addition to being a direct measure of the dimensional stability of the materials. Figure 13 shows the HDT results of neat PBT, the PBT/POE-g-GMA 85/15 blend, and the nanocomposites with MWCNT, ranging from 1 to 5 phr of the nanofiller. It is observed that PBT has an HDT of around 51 °C. With the addition of POE-g-GMA to the PBT matrix, there is a 1 °C decrease in the HDT, reaching 50 °C. This decrease is directly linked to the addition of an elastomeric material to a rigid matrix, thus decreasing the material’s stiffness. With the formation of nanocomposites using MWCNT, there is a considerable increase in the HDT of the materials, reaching values close to 55 °C with the addition of 5 phr of MWCNT. This considerable increase of approximately 5 °C and 4 °C, when compared to the PBT/POE-g-GMA blend and neat PBT, respectively, is directly correlated with the increased stiffness of the nanocomposites, when compared to the precursor materials. Kim [44] studied the effect of MWCNT addition on the properties of PBT and observed that, with increasing MWCNT content, the HDT of the materials increases, which is attributed to the increased flexural stiffness of the studied systems. The HDT results corroborate the findings obtained for impact strength, elastic modulus, and Shore D hardness, in addition to being of great practical relevance, thereby expanding the possibilities of applying the PBT/POE-g-GMA blend in various contexts.

The results obtained from Student’s *t*-test allowed us to evaluate the influence of the incorporation of POE-g-GMA and different MWCNT contents on the HDT of PBT. It was observed that for the PBT/POE-g-GMA blend there is no statistically significant difference compared to PBT, with a *p*-value = 0.0589. On the other hand, the progressive introduction of MWCNTs into the PBT/POE-g-GMA matrix promoted significant changes in the average results, especially from the formulations containing higher contents. The samples with 3, 4, and 5 phr showed statistically significant differences in relation to neat PBT (*p* = 0.0266, 0.0094, and 0.00039, respectively), highlighting the positive effect of MWCNTs on the HDT of the PBT/POE-g-GMA blend on the analyzed property. On the other hand, the formulations with 1 and 2 phr of MWCNT did not show significant differences (*p* > 0.05), suggesting that there is a minimum MWCNT content required for a statistically significant increase in HDT.

### 4.8. Electrical Resistivity and Conductivity

**Figure 14:**
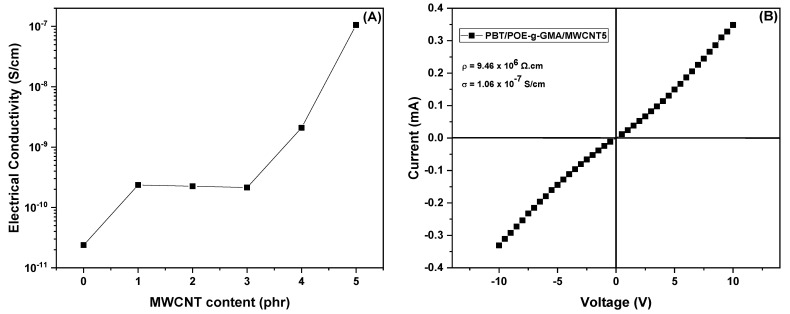
Log σ vs. MWCNT content (**A**) and current–voltage (I–V) curves of the PBT/POE-g-GMA/MWCNT5 (**B**).

Table 3 presents the resistivity and electrical conductivity values obtained for the studied compositions. Figure 14A shows the curve log σ vs. MWCNT content. It can be seen that neat PBT has high resistivity (4.21 × 10^10^ Ω·cm) and low conductivity (2.37 × 10^−11^ S/cm), characteristic of an insulating material. With the addition of the impact modifier POE-g-GMA, there are no significant changes in the electrical properties of the materials, maintaining the same order of magnitude as the neat polymer.

The production of polymer nanocomposites using carbon nanotubes (MWCNTs) aims to form flexible and electrically conductive materials. With the introduction of carbon nanotubes (MWCNTs), a reduction in resistivity and an increase in conductivity are observed. It can be noted that with the addition of 1, 2, and 3 phr of MWCNT, the resistivity decreased by an order of magnitude (10^9^ Ω·cm) compared to neat PBT and the PBT/POE-g-GMA blend, as did the conductivity (10^−10^ S/cm). At higher concentrations (4 and 5 phr), a significant drop in resistivity was observed, reaching 9.46 × 10^6^ Ω·cm for the sample with 5 phr of MWCNT. This result was reflected in an increase of approximately four orders of magnitude in conductivity, reaching 1.06 × 10^−7^ S/cm, suggesting proximity to the electrical percolation threshold. This increase in electrical conductivity with higher MWCNT contents is associated with conductive paths formed by carbon nanotubes dispersed in the polymer matrix, as observed by SEM (Figure 11). Figure 14B represents the current–voltage (I–V) curve for the obtained conductive nanocomposite (PBT/POE-g-GMA/MWCNT5). It can be observed that the material exhibits a practically linear behavior. With increasing current, there is a proportional increase in voltage. This behavior indicates that the analyzed sample follows Ohm’s Law, with constant electrical resistivity within the measured range. Thus, the results of resistivity and electrical conductivity, primarily for higher MWCNT contents (5 phr), combined with the mechanical, thermal, and thermomechanical properties mentioned, can be utilized in products intended for electronic applications, resulting in the formation of a semiconductor material [45]. Furthermore, the literature reports that polymeric materials with electrical conductivity greater than 10^−8^ S/cm [11] can be applied for electrostatic charge dissipation, making the PBT/POE-g-GMA/MWCNT5 nanocomposites promising.

## 5. Conclusions

Nanocomposites of PBT/POE-g-GMA blends (85/15), reinforced with different amounts of carbon nanotubes (MWCNTs) (1–5 phr), were obtained through the melting process. Through the thermal properties, a substantial increase in the crystallization temperature of the nanocomposite materials was observed, when compared to neat PBT, reaching approximately 11 °C. This fact is particularly important from an economic perspective, especially in relation to the process of obtaining injection molded specimens. By FTIR, a decrease in intensity was observed, along with the appearance of a small shoulder, at around 1710 and 1255 cm^−1^, with the addition of POE-g-GMA, indicative of a possible reaction between the blend components. Regarding mechanical properties, the nanocomposites showed substantial improvements in impact strength, regardless of content, compared to neat PBT, reaching values of up to 186 and 156 J/m for 1 and 5 phr of MWCNT, respectively. In addition, tensile strength, elastic modulus, and Shore D hardness were improved compared to the PBT/POE-g-GMA blend. Compared to HDT, there was a gain of approximately 4 °C, resulting in an improvement in the thermomechanical stability of the nanocomposites. Furthermore, obtaining an electrical conductivity of around 10^−7^ S/cm in the nanocomposites reinforced with 5 phr of MWCNT highlights the potential of these materials for applications in sectors that require electrical properties combined with good mechanical strength and thermomechanical stability, as well as in antistatic packaging systems.

## Figures and Tables

**Table 1:** Compositions studied.

Samples	PBT (%)	POE-g-GMA (%)	MWCNT (phr)
PBT	100	-	-
PBT/POE-g-GMA	85	15	-
PBT/POE-g-GMA/MWCNT1	85	15	1
PBT/POE-g-GMA/MWCNT2	85	15	2
PBT/POE-g-GMA/MWCNT3	85	15	3
PBT/POE-g-GMA/MWCNT4	85	15	4
PBT/POE-g-GMA/MWCNT5	85	15	5

phr = parts per hundred of resin.

**Table 2:** Melting for second heating and crystallization parameters obtained through DSC curves.

Samples	*T_m_*_1_ (°C)	*T_m_*_2_ (°C)	Δ*H_m_* (J/g)	*X_c_* (%)	*T_c_* (°C)	Δ*H_c_* (J/g)
PBT	214.8	223.2	34.3	24.5	196.1	37.4
PBT/POE-g-GMA	214.3	222.6	27.3	20.4	194.8	30.2
PBT/POE-g-GMA/MWCNT1	-	222.4	45.8	38.4	203.6	40.5
PBT/POE-g-GMA/MWCNT2	-	221.6	45.6	38.3	204.6	35.7
PBT/POE-g-GMA/MWCNT3	-	222.1	39.6	33.3	206.1	35.4
PBT/POE-g-GMA/MWCNT4	-	221.9	34.1	28.6	206.3	29.2
PBT/POE-g-GMA/MWCNT5	-	222.5	46.3	38.9	207.3	37.9

*T_m_*_1_ first melting peak temperature; *T_m_*_2_ second melting peak temperature; ∆*H_m_* enthalpy of fusion; *X_c_* degree of crystallinity; *T_c_* crystallization temperature; ∆*H_c_* enthalpy of crystallization.

**Table 3:** Resistivity and conductivity results of the studied samples.

Samples	Resistivity (Ω·cm)	Conductivity (S/cm)	Conductivity (S/m)
PBT	4.21 × 10^10^	2.37 × 10^−11^	2.37 × 10^−9^
PBT/POE-g-GMA	2.11 × 10^10^	4.74 × 10^−11^	4.74 × 10^−9^
PBT/POE-g-GMA/MWCNT1	4.25 × 10^9^	2.35 × 10^−10^	2.35 × 10^−8^
PBT/POE-g-GMA/MWCNT2	4.44 × 10^9^	2.25 × 10^−10^	2.25 × 10^−8^
PBT/POE-g-GMA/MWCNT3	4.68 × 10^9^	2.14 × 10^−10^	2.14 × 10^−8^
PBT/POE-g-GMA/MWCNT4	4.79 × 10^8^	2.08 × 10^−9^	2.08 × 10^−7^
PBT/POE-g-GMA/MWCNT5	9.46 × 10^6^	1.06 × 10^−7^	1.06 × 10^−5^

## Data Availability

The data supporting the findings of this study are available from the corresponding author upon reasonable request.
